# Impact of levetiracetam use in glioblastoma: an individual patient-level meta-analysis assessing overall survival

**DOI:** 10.1007/s10143-024-03137-x

**Published:** 2024-12-09

**Authors:** Martin Vychopen, Agi Güresir, Alim Emre Basaran, Erdem Güresir, Johannes Wach

**Affiliations:** Department of Neurosurgery, University Hospital Leipzig, Liebigstraße 20, Leipzig, 04103 Germany

**Keywords:** Anti-epileptic drug, Glioblastoma, Individual Patient Data, Levetiracetam, Overall survival

## Abstract

**Background:**

Levetiracetam (Lev), an antiepileptic drug (AED), enhances alkylating chemotherapy sensitivity in glioblastoma (GB) by inhibiting MGMT expression. This meta-analysis evaluates Lev's impact on GB treatment by analyzing overall survival of individual patient data (IPD) from published studies.

**Methods:**

IPD was reconstructed using the R package *IPDfromKM*. Pooled IPD Kaplan–Meier charts of survival stratified by Lev therapy were created using the R package Survminer. One- and two-stage meta-analyses of Lev treatment regarding survival was performed.

**Results:**

Three articles covering 825 patients were included out of 3567 screened records. Lev usage prevalence was 0.36. IPD from 590 IDH wild-type glioblastomas, with a median follow-up of 16.1 months, were utilized. Pooled data revealed median survival times of 19.2 months (95%CI: 16.4–22.0) for Lev users versus 16.5 months (95%CI: 15.2–17.8) for partial/no use (*p* = 0.006). One-stage meta-analysis indicated a significant association between Lev use and survival in IDH wild-type GB (HR: 1.33, 95%CI: 1.08–1.64, *p* = 0.007). Two-stage meta-analysis confirmed these results.

**Conclusions:**

This meta-analysis highlights that Lev use may prolong survival in IDH wild-type GB patients. Further randomized trials are needed to confirm these findings and identify subgroups benefiting most from Lev treatment.

**Supplementary Information:**

The online version contains supplementary material available at 10.1007/s10143-024-03137-x.

## Introduction

Glioma-associated epilepsy represents an additional burden for glioblastoma (GB) patients. Up to 50% of patients might present themselves with epileptic seizures (ES) [1]. The exact mechanism of seizure development remains unclear, as different pathophysiological mechanisms were proposed. These include enhanced neuronal plasticity which might result in seizure development, or unfavorable anatomical localization of the GB irritating epileptogenic areas, mainly in frontotemporally localized tumors [2, 3].

However, patients with ES might receive earlier therapy of the tumor, which might indirectly result in better overall survival (OS) compared to patients without ES, who receive the therapy at later stages of the disease [4]. This might indirectly explain the highly debated positive effect of epilepsy on OS in GB patients.

In case of therapy-refractory seizures, quality of life is significantly reduced and the potentially resulting reduced patient´s physical functional status can lead to a therapy-limiting situation in terms of this incurable disease [5, 6]. Although some authors argue that the low incidence of postoperatively developed seizures does not justify prophylactic administration of antiepileptic drugs (AED), there is still no clear consensus on the use of AED in patients with brain tumors [5, 7]. The current EANO practice guideline does not recommend prophylactic administration of AED drugs even in such cases who undergo awake craniotomy [8]. Patients with continuously administered AED might develop side effects including severe depression, anxiety, and fatigue [1]. If combined with temozolomide, AED administration might increase the cumulative hematotoxic effect of the therapy [9].

Nevertheless, several studies showed that levetiracetam (Lev), if used continuously, might paradoxically enhance the OS of patients undergoing radiochemotherapy despite the epilepsy burden [9–12]. This clinically apparent effect might be explained by experimentally validated inhibitory effect of LEV on O6-methylguanine-DNA methyltransferase (MGMT) and subsequent sensitization of GB-cells to temozolomide therapy [13]. A conventional meta-analysis conducted on the treatment effect measures (Hazard ratio) of Lev administration on OS supports a positive effect of LEV administration in patients with unmethylated MGMT-promoters high-grade gliomas (IDH mutant or IDH wild-type) [14].

Up to date, an individual patient data (IPD) meta-analysis of Lev administration in GB patients has not been performed yet. The aim of our study is to analyze the impact of Lev on OS and additionally stratifying the use of Lev treatment during chemoradiotherapy in IDH wild-type GB patients only in order to be in line with the current 2021 WHO CNS classification [15].

## Methods

This systematic review and meta-analysis adhered to the guidelines outlined in the Preferred Reporting Items for Systematic Reviews and Meta-Analyses (PRISMA) statement (Supplementary Fig. 1). The study protocol was prospectively registered in the 'International Prospective Register of Systematic Reviews' (PROSPERO, Registration ID: CRD42024507697) [16].

### Search strategy and study inclusion criteria

We conducted a literature search in the four databases Pubmed, Medline, Cochrane and Embase database for all clinical studies regarding Lev treatment in glioblastoma up to January 26, 2024. Studies published in English were retrieved. The search strategy was conducted based on the PICOS criteria [17]. The following mesh terms were used to identify eligible studies: 1) “glioblastoma “AND “levetiracetam”; 2) “glioma “ AND “levetiracetam “ (see supplementary Table 1). Inclusion criteria required data on duration of levetiracetam treatment, adjuvant radiochemotherapy with temozolomide, primary diagnosis of GB, and follow-up data regarding OS displayed in Kaplan–Meier charts with number at risk tables. Included were all two-arm and multi-arm studies reporting on OS of patients with glioblastoma and homogenously administered radiochemotherapy with temozolomide, with the intervention being the administration of LEV during radiochemotherapy. Control group was represented by patients who received radiochemotherapy with temozolomide only. Groups with additional chemotherapeutic drugs administered during the treatment were excluded, as this could severely bias the potential effect of the analyzed drug Lev on OS. Two reviewers (JW, MV) independently screened abstracts, and full-text articles for two rounds, with any residual conflicts resolved by a supervising third reviewer (EG).

### Quality assessment

The assessment of quality and risk of bias was conducted using the National Institutes of Health Quality Assessment Tool for observational cohort and cross-sectional studies (NIH-QAT) [18].

### Data extraction

Two reviewers (MV, JW) independently extracted the following characteristics from the publications: year of publication, country, study timeframe, applied version of WHO classification system for CNS tumors, total number of patients, age, sex, MGMT promoter status, IDH-1/2 status, extent of resection, adjuvant therapy, and median survival times. The IPD for survival analysis was obtained by digitizing the published Kaplan–Meier survival curves and number-at-risk tables using DigitizeIt (Version 2.5.10, Germany) [19]. This process was carried out for GB patients with continuous Lev use, partial or no Lev use in the identified articles. The extracted information on survival and the published number-at-risk-tables were utilized to reconstruct the Kaplan–Meier curves for each included study, following the method outlined by Liu et al. [20] with the R package IPDfromKM in R (Version 4.3.1, R Foundation for Statistical Computing, Austria). Comparisons were made between the reconstructed curves, risk tables, estimated hazard ratios (HRs), and estimated 95% confidence intervals (CI) with the corresponding data in the original publications. In cases of apparent discrepancies, the information extraction process was repeated.

### Statistics

The IPD information of all survival time data from all the included studies was pooled, and Kaplan–Meier charts of OS were created using the R packages Survminer and Survival. The 6-, 12-, 18-, and 24-months survival rates were constructed. The hazard ratios of each included investigation as well as the pooled HR and corresponding 95% CI between patients with Lev treatment and those without were calculated. In the two-stage meta-analysis the estimated HRs and the corresponding 95% CIs of each study were combined using fixed effect model with the generic inverse variance method in case of low heterogeneity (< 40%) [21].

The hazard ratios were estimated and then transformed into natural logarithms (LN). The standard errors (SE) for each study were computed from the 95% confidence interval (CI) using the formula: SE = (LN(upper CI limit)–LN(lower CI limit))/3.92, in accordance with the guidelines provided in the Cochrane Handbook for Systematic Reviews of Interventions, Version 6.4 [21]. The significance of each study's relative contribution, determined by its sample size, was considered when estimating treatment effects. The combined estimates were visually presented in forest plots using the R package *Metafor*. Statistical significance was defined as *P* < 0.05. Publication bias was visually examined using funnel plots with the R package *Metafor*.

## Results

### Study selection and study characteristics

**Fig. 1:**
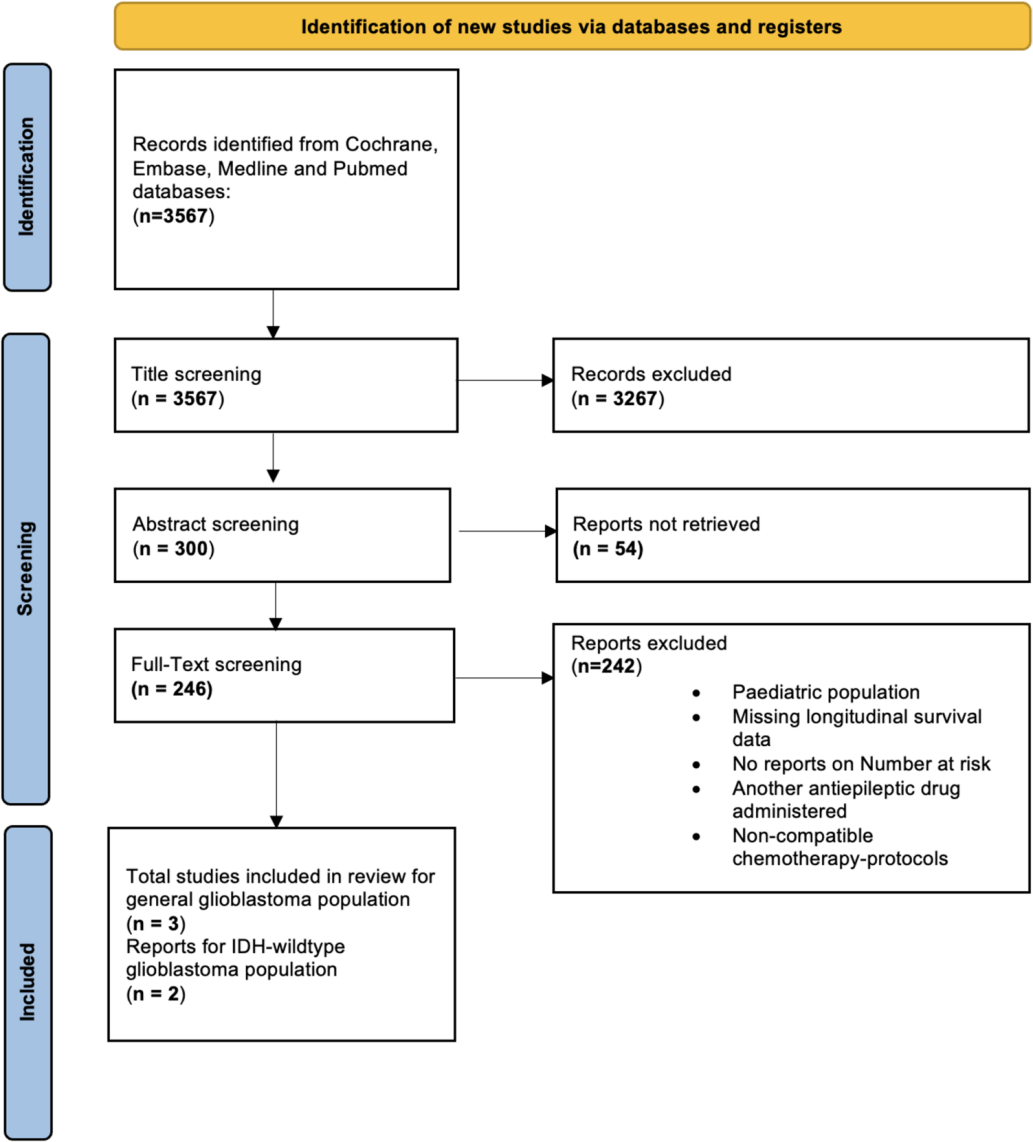
Prisma flow diagram illustrating the study selection process

We found 3567 studies being eligible for screening. After title screening and abstract screening, 246 studies remained for full-text screening. Excluded were all studies with pediatric patients, no data on long term outcome or number at risk and studies reporting on antiepileptic drug different than LEV. After reviewing 246 Studies, 3 articles were included in the cumulative GB cohort and 2 were included in the analysis of IDH-wildtype GBs (see Fig. 1 displaying the PRISMA flow diagram).

To assess for the IDH-wildtype differences, 2 separate analyses were performed: Patients with IDH-wildtype glioblastoma (2 Studies) and all available GB patients (3 Studies). Both cohorts are reported on separately. The characteristics of the included studies are presented in Table 1.

**Table 1:** Characteristics of studies included in IPD meta-analysis

Study	Year	Country	Reported Timeframe	WHO Classi-fication	Number of patients (n)	IDH Status	OS (Median, months)
Bianconi et al. [20]	2024	Italy	2015 – 2021	WHO CNS 2021 tumor classification	130	IDH-Wildtype	15.64
Pallud et al. [21]	2022	France	2010 – 2018	WHO CNS 2016 tumor classification with retrospective histological reassesment	460	IDH-Wildtype	17.40
Rigamonti et al. [22]	2018	Italy	2004—2017	N/A	235	N/A	11.00

The present meta-analysis includes 825 patients. The study of Bianconi et al. [22] presents a single-center observational cohort of IDH-wildtype GB patients who underwent surgical resection. Patients who underwent stereotactic biopsy only, discontinued radiochemotherapy, or primarily received palliative care therapy were excluded. Among the remaining cohort, 101 patients exhibited MGMT-promotor methylation.

The study of Pallud et al. [23] presents an observational single-institutional cohort of IDH-wildtype GB patients. There were no exclusion criteria applied to the extent of the surgical procedure, meaning that patients undergoing stereotactic biopsies were included.

Rigamonti et al. [24] performed a multicentric retrospective study of 285 glioblastoma patients. Similar to Pallud et al. [23] all extents of resection are represented in this cohort. Contrary to Bianconi et al., discontinuation of the radiochemotherapy or primary palliative care was not an exclusion criterium. Furthermore, the study by Rigamonti et al. [24], published in 2018, adhered to the previous WHO classification system, which implies that there are also potential previously defined IDH-mutant GBs. Hence, this study was excluded in the IPD survival analysis of IDH wild-type GBs only.

All three studies reported Kaplan–Meier charts and number at risk tables regarding the OS probabilities of patients undergoing Lev administration compared to those without. Supplementary Table 2 summarizes the cohort characteristics of the individual studies.

### Reconstructed pooled survival curves and one-stage meta-analysis of the impact of levetiracetam use in high-grade glioma

Three articles encompassing 825 patients were deemed eligible for inclusion. The prevalence of Lev use was 0.36 (294/825). These three studies encompassed two studies investigating exclusively IDH wild-type glioblastoma [22, 23], and one study using the previous WHO classification system which potentially also included IDH mutant tumors [24]. The reconstructed OS curves and side-by-side comparisons with the original plots were performed. The estimated HRs and corresponding 95% CI of included studies in the one-stage analysis are displayed in supplementary Table 3.

**Fig. 2:**
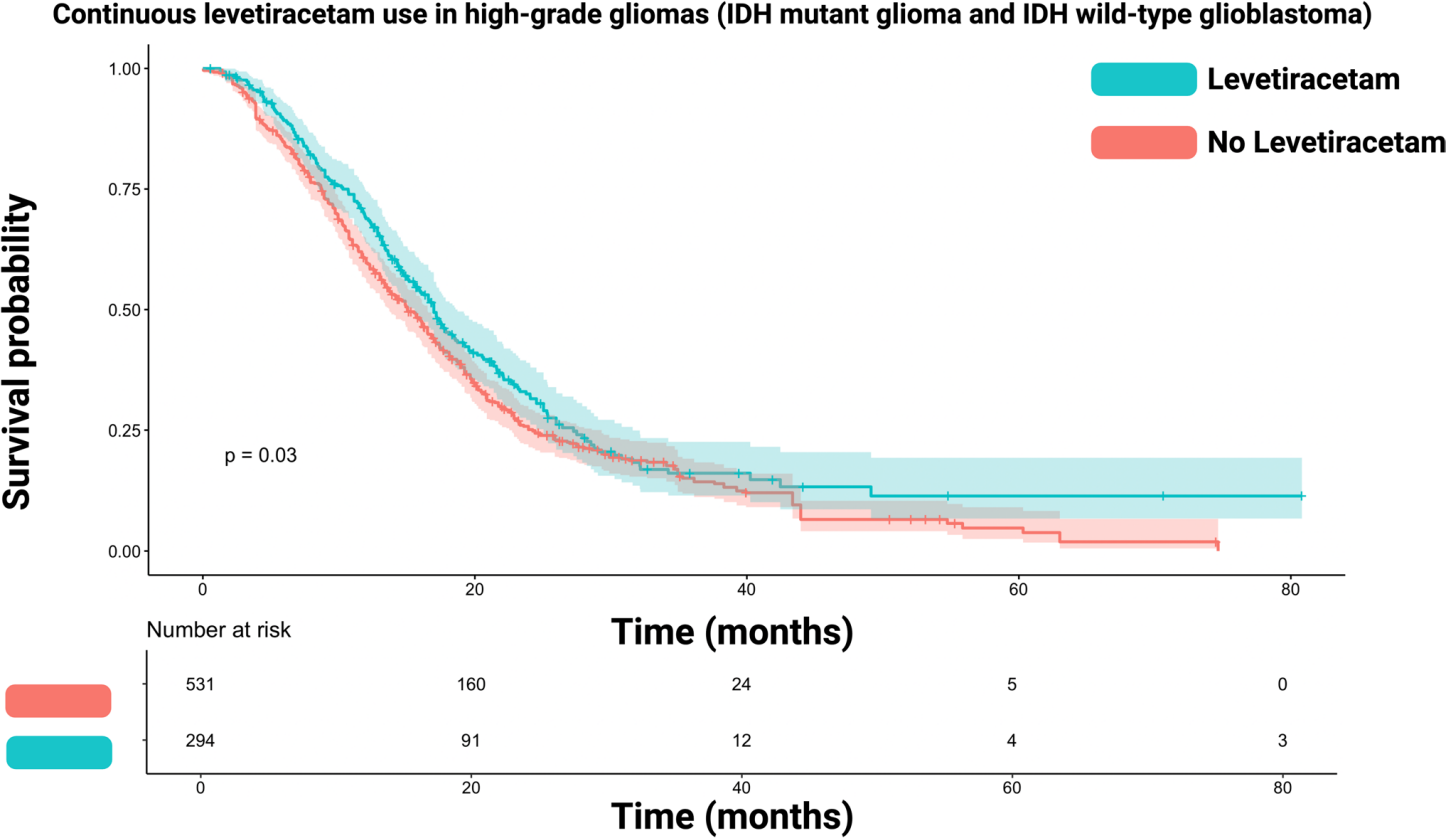
Kaplan–Meier chart displaying survival probability stratified by continuous levetiracetam use (turquoise) and no/partial levetiracetam use (red) in high-grade gliomas. The log-rank test (*p* = 0.03) showed a significantly enhanced survival time in those patients with continuous levetiracetam use. The shadowed areas surrounding the plots display the confidence intervals

All the reconstructed Kaplan–Meier plots in each of the studies, along with published plots, displayed remarkable similarity, and any disparities in the number-at-risk tables were minimal. The median (IQR) follow-up time for OS of the reconstructed IPD was 14.6 months (8.5–22.3). Figure 2 illustrates the reconstructed OS curve for the total cohort (GB patients including IDH mutant cases from Rigamonti et al. [24]). The survival analysis was stratified by continuous Lev use or no/partial Lev use. The median survival time in those with continuous Lev use was 17.0 (95%CI: 15.5–18.5) months, whereas those without or only partial Lev use had a median survival time of 15.0 (95%CI: 13.7–16.4) months (log-rank test: *p* = 0.03).

The Hazard Ratio of continuous Lev use in the entire high-grade glioma IPD cohort for enhanced survival time was 1.20 (95%CI: 1.02–1.42, *p* = 0.03).

### Reconstructed pooled survival curves and one-stage meta-analysis of the impact of levetiracetam use in IDH wild-type glioblastoma

**Fig. 3:**
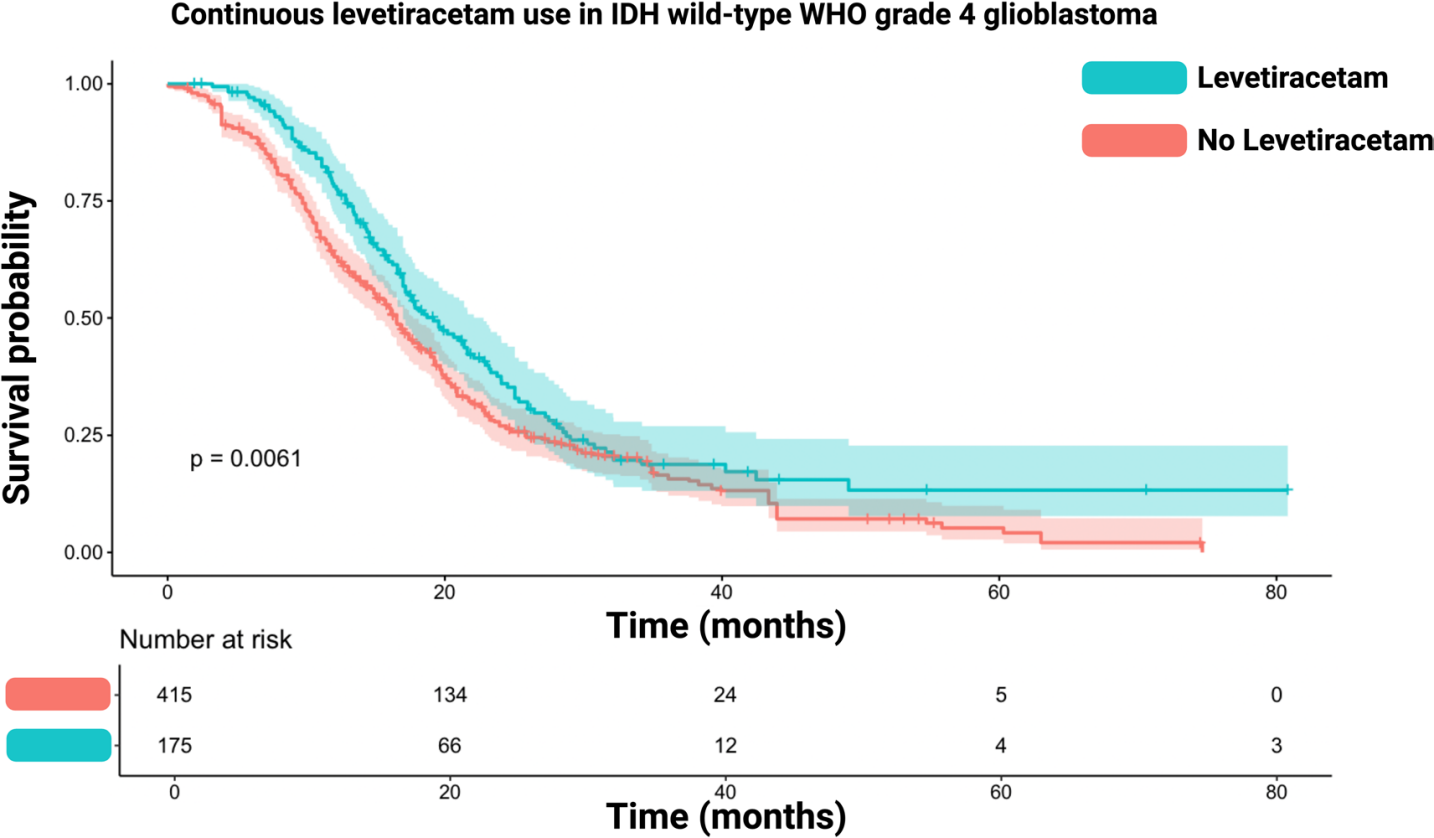
Kaplan–Meier chart sowing survival probability stratified by continuous levetiracetam use (turquoise) and no/partial levetiracetam use (red) in IDH wild-type WHO grade 4 glioblastomas. The log-rank test (*p* = 0.0061) showed a significantly enhanced survival time in those patients with continuous levetiracetam use. The shadowed areas surrounding the plots display the confidence intervals.greater the weight of the study. The diamond corresponds to the logHR of the pooled data

The reconstructed survival times from IPD were further pooled in an exclusively IDH wild-type GB cohort (*n* = 590) being in line with the present WHO classification system [15]. Figure 3 illustrates the reconstructed OS curve for the IDH wild-type GB cohort [22, 23]. The survival analysis was stratified again by Lev use during radiochemotherapy or no/partial Lev use. The median survival time in those with continuous Lev use was 19.2 (95%CI: 16.4–22.0) months, whereas those without or only partial Lev use had a median survival time of 16.5 (95%CI: 15.2–17.8) months (log-rank test: *p* = 0.006). The 6, 12-, 18-, and 24-month survival rates for IDH wild-type GB patients with continuous Lev use were 97.1%, 78.1%, 52.1% and 36.7.%. The group of IDH wild-type GB patients without or only partial Lev use had 6, 12-, 18-, and 24-month survival rates of 88.6%, 64.0%, 43.6%, and 26.9%. The Hazard Ratio of continuous Lev use in the IDH wild-type GB IPD cohort for enhanced survival time in the one-stage meta-analysis was 1.33 (95%CI: 1.08–1.64, *p* = 0.007).

### Two-stage meta-analysis of overall survival in IDH wild-type glioblastomas

**Fig. 4:**
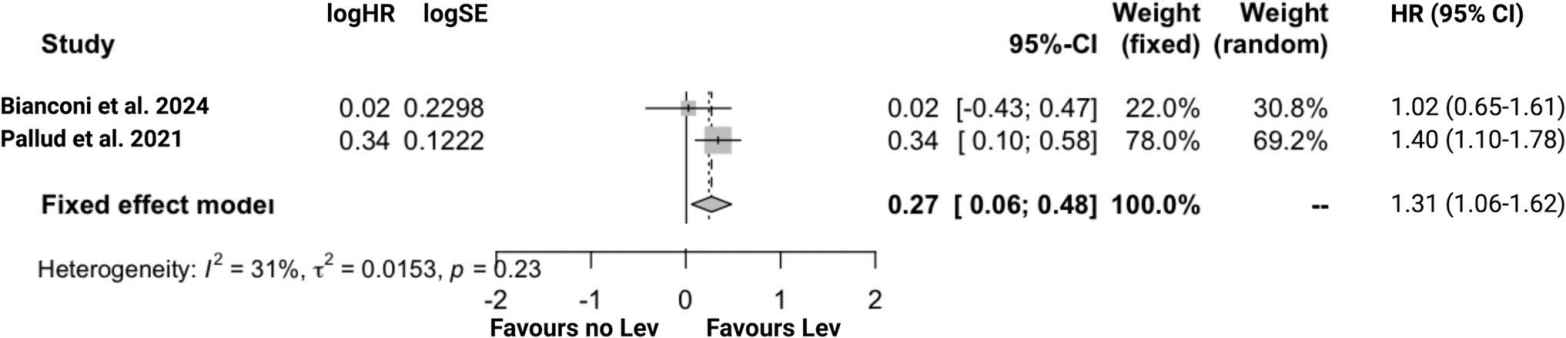
Forest plot displaying log (Hazard ratio), log (Standard error), HR, and 95% CI estimates for OS in a fixed effect with the inverse variance method of the included studies for analysis of IDH wild-type WHO grade 4 glioblastoma. X-axis locations of squares label the hazard ratio. The weight of the included investigations is also reported. The diamonds constitute the hazard ratios of the pooled cohort in each model

To validate the robustness of the findings, a two-stage meta-analysis was conducted. Concerning overall survival, the combined hazard ratio 1.31 (95%CI: 1.06–1.62) in the fixed-effect model corroborates the results of the one-stage meta-analysis, affirming an association between continuous Lev use and enhanced survival time in IDH wild-type glioblastoma.The assessment indicated no substantial heterogeneity (*I*^2^ = 31%, *p* = 0.23), which makes the results from the fixed-effect model appropriate. Figure 4 displays Forest plots summarizing the two-stage analyses of survival time in IDH wild-type glioblastoma.

### Bias and quality evaluation

To ascertain a reliable evaluation, multiple measures were employed to scrutinize the potential presence of publication bias. Initially, an exhaustive literature search strategy was deployed. Subsequently, the studies incorporated into this meta-analysis rigorously adhered to pre-established inclusion and exclusion criteria. Lastly, the assessment of publication was conducted through the examination of funnel plots (refer to supplementary Fig. 2) pertaining to the survival analysis. The NIH-QAT tool was utilized to assess quality, resulting in favorable ratings for the included studies. The ratings for each of the 14 NIH-QAT domains can be found in supplementary Fig. 3. Two of the studies showed discrepancies in recruitment criteria regarding the extent of resection, which also was not statistically analyzed. None of the included studies reported on the level of exposure in terms of Lev dosage. Blinding of patients or physicians was not possible in the retrospective setting.

## Discussion

In the present meta-analysis, we investigated the effect of continuous Lev therapy in high-grade gliomas with special focus on the survival time in IDH wild-type GB. Generally, the overall survival time in IDH wild-type GB was found to be enhanced by the continuous use of Lev (see supplementary Fig. 4). The present investigation encompasses the first pooled analysis of longitudinal survival data from 590 IDH wild-type WHO grade 4 GB patients and is the largest survival analysis of continuous Lev use so far. Furthermore, the present cohort encompasses IDH wild-type GB patients, who underwent the standard chemoradiation protocol using temozolomide.

Median survival time in those IDH wild-type GB patients with continuous Lev use was 19.2 months, whereas those without or only partial Lev use had a median survival time of 16.5 months. This finding is in line with a previous conventional meta-analysis of treatment effect measures (Hazard ratio), which found that preoperative Lev administration was associated with longer survival [25]. Furthermore, there is also data from another conventional meta-analysis, which pooled the Hazard ratios of survival from 11 studies conducted between 2012 and 2021, which showed only a trend towards an improved survival time in patients receiving Lev treatment (HR: 0.89, 95% CI: 0.78–1.02, *p* = 0.09) [14]. However, the present meta-analysis focuses on IDH-wild-type GBs which constitutes the current WHO classification system [15].

In vitro investigations have indicated that Lev exerts a sensitizing effect on GB cells in the presence of temozolomide, primarily attributed to the reduction of O-6-methylguanine-DNA methyltransferase (MGMT) protein expression [26]. The molecular mechanism underlying this phenomenon involves the upregulation of histone deacetylase 1 (HDAC1) transcription by levetiracetam and the recruitment of HDAC1/mSin3A multiprotein corepressor complex to the p53-binding site within the MGMT promoter [13, 26, 27]. Furthermore, Bobustuc et al. [13] highlighted that the suppression of MGMT expression by Lev is contingent upon on the presence of p53, mSin3A, and HDAC1. This implies that individuals with diminished p53 viability, like males, may not be well-positioned to derive advantages of Lev treatment.

Furthermore, the combined administration of Lev and temozolomide demonstrates an augmented anti-glioblastoma functioning, resulting in tumor suppression by inducing senescence in GB cells and activating the apoptotic pathway [27]. Neuronal activity, mediated through functional neurogliomal chemical excitatory glutamatergic synapses involving AMPA receptors between presynaptic pyramidal neurons and postsynaptic glioma cells, has been identified in high-grade diffuse gliomas [28, 29]. The induced release of calcium, triggered by neuronal activity (i.e., periodic calcium activity), extensively fosters the progression of glioma [30]. Given that Lev exhibits anti-AMPA effects, its prolonged usage might further impact the neuronal-glial interaction [31]. This, in turn, potentially enhances the inhibition of glioma progression, generating vulnerability for treatment. The comprehensive understanding of these complex molecular and synaptic interactions is of paramount importance regarding a potential future individualized therapy (see supplementary Fig. 5).

The present results conflict the findings from Happold et al. [32], which investigated 1869 patients with the primary diagnosis of a GB from the results of four randomized clinical studies. However, this study analyzed various oncological therapies (e.g., temozolomide only, cilengitide, bevacizumab), and was in line with the old WHO classification system. Therefore, we have excluded this study to be in line with our predefined selection criteria of GBs being treated by temozolomide only. As far as the clinical implications of the present analysis are concerned, Lev therapy may have beneficial effect on the tumor therapy in addition to the standardized combined radiochemotherapy with temozolomide in IDH wild-type 4 GBs. Nevertheless, the implementation of Lev therapy in the absence of symptomatic epilepsy has to be weighted up against the potential side effect profile of Lev (e.g., psychiatric side effects) [33]. Furthermore, perioperative Lev therapy was also suggested to negatively influence the intraoperative protoporphyrin IX accumulation in 5-aminolevulinic acid-guided GB surgery [34]. Future studies will also have to analyze whether the initiation of a continuous Lev therapy has also effects on the survival time measured from the timepoint of the diagnosis of a recurrent GB because the present investigation investigates only newly diagnosed GB.

### Strengths and limitations

The present meta-analysis is the first investigation of longitudinal IPD of 590 IDH wild-type GB patients showing a potential use of continuous Lev treatment in these patients in addition to the conventional adjuvant radiochemotherapy with temozolomide. Although IPD meta-analysis is considered the gold standard for longitudinal time-to-event data [21], we were unable to further stratify the IPD by crucial variables such as extent of resection, MGMT status, and Karnofsky performance status [35]. For instance, the study by Pallud et al. [23], which showed the strongest effect of Lev therapy included only 47.2% of patients who underwent a gross total resection, whereas in the study by Bianconi et al. [22] 89% of the patients underwent a complete resection of the contrast-enhancing tumor portion. These factors (MGMT & extent of resection) might be confounding biases impacting the endpoint parameters. Additionally, a major limitation of this study is that all included investigations are of a retrospective nature, which may influence the reliability of the findings. The present meta-analysis includes two studies in publication bias analysis, which implicates the risk of publication bias [36, 37]. Methods like funnel plot asymmetry might be unreliable for this small number studies, making it difficult to draw definitive conclusions.

A notable constraint is also the potential presence of protopathic bias. In such instances, a patient receiving Lev treatment and another one without it might exhibit comparable survival rates from tumor inception to timepoint of death. Furthermore, potential dose-dependent effects of Lev is unknown. Nevertheless, the individual using Lev may be recorded as having extended clinical survival due to the necessity of managing seizures with Lev, which could have led to an earlier confirmation of the diagnosis as a GB.

## Conclusions

The present meta-analysis is the first investigation using reconstructed IPD to reveal the effect of Lev therapy during radiochemotherapy in IDH wild-type WHO grade 4 glioblastoma. The results indicate that Lev therapy might be associated with prolonged survival. Further prospective randomized data are needed to validate these findings and identify the optimum molecular subgroup of GB patients benefiting most from Lev therapy.

## Supplementary Information

Supplementary Material 1.

Supplementary Material 2.

Supplementary Material 3.

Supplementary Material 4.

Supplementary Material 5.

Supplementary Material 6.

Supplementary Material 7.

Supplementary Material 8.

## Data Availability

No datasets were generated or analysed during the current study.
