# Supplementary material for: Impact of levetiracetam use in glioblastoma: an individual patient-level meta-analysis assessing overall survival
Source: Neurosurg Rev. 2024 Dec 9;47(1):897. doi: 10.1007/s10143-024-03137-x (PMC11628436; doi:10.1007/s10143-024-03137-x)
Supplement: Supplementary file 1 — Supplementary Material 1. [file 10143_2024_3137_MOESM1_ESM.pdf]

# PRISMA 2020 Checklist

| Section and Topic | Item # | Checklist item | Location where item is reported / Page No. |
|---|---|---|---|
| TITLE | | | |
| Title | 1 | Impact of continuous levetiracetam use in glioblastoma: An Individual Patient-Level Meta-Analysis Assessing Overall Survival | Title / 1 |
| ABSTRACT | | | |
| Abstract | 2 | **BACKGROUND:** Levetiracetam (Lev), an antiepileptic drug (AED), exerts its influence on glioblastoma (GB) by sensitizing the tumor to alkylating chemotherapy through the inhibition of MGMT expression. Augmenting the clinical therapy for GB with Lev presents a potential avenue for optimizing temozolomide therapy. This meta-analysis systematically analyzes the literature and reconstructs survival probabilities of individual patient data (IPD) stratified by Lev use. **METHODS:** IPD of overall survival (OS) were extracted from published Kaplan-Meier charts using the R package *IPDfromKM* in R (Version 4.3.1, The R Foundation for Statistical Computing). Reconstructed Kaplan-Meier Charts of the pooled IPD data were generated. One-stage and two-stage meta-analyses were conducted. Hazard ratios (HR) served as pertinent metrics for assessing effectiveness. **RESULTS:** Out of 3567 records screened, three articles encompassing 825 patients were deemed eligible for inclusion. The prevalence of continuous Lev use was 0.36 (294/825). IPD data with a median follow-up of 16.1 months from 590 IDH-1 wild-type glioblastomas were created. Reconstructed OS curves for the pooled cohort revealed median survival times of 19.2 (95% CI: 16.4-22.0), and 16.5 (95% CI: 15.2-17.8) months for IDH-1 wild-type WHO grade 4 GB patients with continuous Lev use, and partial/no Lev use, respectively ($p$ = 0.006). The one-stage meta-analysis demonstrated an association between continuous Lev use and survival time in IDH-1 wild-type GB (HR: 1.33, 95% CI: 1.08-1.64, $p$ = 0.007). The two-stage meta-analysis validated the findings of the one-stage analysis regarding survival time. **CONCLUSIONS:** The present meta-analysis from IPD underscores that continuous use of Lev may extend the survival time of GB patients with IDH-1 wild-type. The critical imperative now lies in the conduction of a randomized trial to validate these results and pinpoint the specific subgroups of GB patients benefiting most from Lev treatment. | Abstract / 2 |
| INTRODUCTION | | | |
| Rationale | 3 | Levetiracetam (Lev) is an antiepileptic drug which potentially sensitizes glioblastoma (GB) to alkylating chemotherapy via the MGMT pathway. Hence, there is an increasing interest regarding levetiracetam treatment in the field of GB to increase the efficacy of temozolomide. | Introduction / 3 |
| Objectives | 4 | This meta-analysis systematically analyzes the literature and reconstructs survival probabilities of individual patient data (IPD) stratified by continuous Lev use. | Introduction / 3 |
| METHODS | | | |
| Eligibility criteria | 5 | We searched for 1) “glioblastoma“ AND “levetiracetam”; 2) “glioma“ AND “levetiracetam“in Pubmed, Medline, Cochrane and Embase database until 26th January 2024 and found three studies being eligible for inclusion. Subsequently, we screened meta-analyses on Lev for further possibly eligible studies. Included were all studies describing survival data stratified by continous Lev use or not with Kaplan-Meier charts and corresponding patient number at risk tables. We excluded case studies, study protocols, non-clinical trials and meta-analyses.. Two reviewers (JW, MV) independently screened abstracts, and full-text articles for two rounds, with any residual conflicts resolved by a third reviewer (EG). | Methods / 4 |
| Information sources | 6 | We searched for 1) “glioblastoma“ AND “levetiracetam”; 2) “glioma“ AND “levetiracetam“in Pubmed, Medline, Cochrane and Embase database until 26th January 2024 and found three studies being eligible for inclusion. | Methods / 4 |
| Search strategy | 7 | We conducted a literature search in Pubmed, Medline, Cochrane and Embase database for all clinical studies including following terms: “glioblastoma“, “glioma“ and “levetiracetam“. Subsequently, we screened already available Meta-analyses for additional studies. | Methods / 4 |

# PRISMA 2020 Checklist

| Section and Topic | Item # | Checklist item | Location where item is reported / Page No. |
|---|---|---|---|
| | | | |
| Selection process | 8 | Excluded were all studies with pediatric patients, studies which did not reported on long term outcome or did not report on number at risk and studies reporting on antiepileptic drug different than LEV. Included were all two-arm and multi-arm studies reporting on OS of patients with glioblastoma and homogenously administered radiochemotherapy, with the intervention being the continuous administration of LEV. Control group was represented by patients who received radiotherapy and chemotherapy with temozolomide solely. Groups with additional chemotherapeutic drugs administered during the course of the treatment were excluded, as this could severely bias the levetiracetam effect on overall survival. | Methods / 4 |
| Data collection process | 9 | The data collection was performed by two authors independently (MV, JW). First the study titles were screened, then the corresponding abstract and in case of further uncertainty the full-text was screened by two authors (JW and EG) until all retrieved studies were either included or excluded. Two authors (JW, MV) independently extracted the following data from the studies: clinical and molecular characteristics of GB patients. We extracted and combined the IPD for overall survival using the Kaplan-Meier plots and the hazard ratio of the included studies. The data-reconstruction was performed with the R package *IPDfromKM* in R software version 4.3.1 (R Foundation for Statistical Computing, Vienna, Austria). | Methods / 4-5 |
| Data items | 10a | We analyzed the studies to conduct a meta-analysis according to the following outcomes:<br>1) In case of levetiracetam use, following outcomes were analyzed: Probabilities of survival in continuous levetiracetam use or without/partial levetiracetam use | Methods / 4-5 |
| Study risk of bias assessment | 11 | The National Institutes of Health Quality Assessment Tool for observational cohort and cross-sectional studies (NIH-QAT) was used for the assessment of quality and risk of bias of included studies. | Methods / 4-5 |
| Effect measures | 12 | We measured and reported outcomes in Forest-plots, providing heterogeneity and inconsistency analysis, pooled hazard ratios and statistical significance. Furthermore, a pooled Kaplan-meier chart of the reconstructed IPD was generated. | Methods / 5 |
| Synthesis methods | 13a | To see all the eligible studies and reported outcomes, see Table 1 | Methods / 5 |
| | 13b | To see all the eligible studies and reported outcomes, see Table 1 | Methods/ 5 |
| | 13c | For visualization of our meta-analysis, Forest Plots were created with the R package *Metafor*. IPD was generated using the R package *IPDfromKM* in R software version 4.3.1 (R Foundation for Statistical Computing, Vienna, Austria) [11]. Kaplan-Meier curves of overall survival were constructed for the whole included patient cohort using the R package *Survminer* and *Survival* in R software version 4.3.1 (R Foundation for Statistical Computing, Vienna, Austria). | Methods / 5 |
| | 13d | Patient demographics and disease-specific characteristics of the included studies were recorded. The IPD information of all survival data from all the included trials was pooled, and Kaplan-Meier curves of overall survival were constructed for the whole included patient cohort using the R package *Survminer* and *Survival* in R software version 4.3.1 (R Foundation for Statistical Computing, Vienna, Austria). The 6-, 12-, 18- and 24-months survival probabilities were calculated. The hazard ratios (HR) of each individual study as well as the pooled HR and corresponding 95% confidence intervals (CI) between those with continuous Lev treatment and those without/partial Lev use were estimated. | Methods / 5 |

# PRISMA 2020 Checklist

| Section and Topic | Item # | Checklist item | Location where item is reported / Page No. |
|---|---|---|---|
| | | In the two-stage meta-analysis, we combined the estimated hazard ratios (HRs) and their corresponding 95% CI from individual studies using a random-effects model, employing the generic inverse variance method. The calculated HRs were logarithmically transformed (LN). For each study, the standard error (SE) was derived from the 95% CI using the formula: SE = (LN(upper CI limit) – LN(lower CI limit)) / 3.92, as outlined in the Cochrane Handbook for Systematic Reviews of Interventions, Version 6.4 [12]. Heterogeneity across the included studies was evaluated using $I^2$ statistics, with a threshold of >50% displaying substantial heterogeneity [13]. The aggregated results were presented in forest plots utilizing the R package *Metafor*. Weight of the relative contribution of the included studies, based on the sample size, was considered concerning the estimation of the treatment effects. A significance level of $p < 0.05$ was considered statistically significant. To evaluate publication bias, we created funnel plots with the R package *Metafor*. Visual assessment via funnel plots was performed. | |
| | 13e | Not available | NA |
| | 13f | Not available | NA |
| Reporting bias assessment | 14 | According to our strict inclusion criteria and high quality of included studies, we do not suppose to have bias due to missing results. Risk of bias assessment was performed for all included studies | Methods / 4-5 |
| Certainty assessment | 15 | Quality assessment was performed and the risk of bias assessment is summarized in supplemental figure 2. | Methods / 4-5 |
| RESULTS | | | |
| Study selection | 16a | Process of the search and selection is summarized in Figure 1. | Figure 1 / 6 |
| | 16b | Included studies are summarized in table 1. | Figure 1 / 6 |
| Study characteristics | 17 | Characteristics of included studies are summarized in table 1. | Table 1 / 6 |
| Risk of bias in studies | 18 | Risk of bias analysis is reported in supplementary figure 2. | Supplemental Figure 2-3 / 8 |
| Results of individual studies | 19 | Analysis of each outcome are separately reported on with it´s own forest plot in Table 2 and Figure 4. | Figures 4 and table 2 / 6-8 |
| Results of syntheses | 20a | Risk of bias assessment is summarized in the supplementary figure 2. Figure 5 represents the funnel plot visualizing the publication bias | Suppl. Figure 2 & Figure 3 / 8 |
| | 20b | Results of the statistical syntheses are summarized in the Figures 2, Figure 3, and Figure 4. | Figures 2-4 / 6-8 |
| | 20c | Results regarding heterogeneity are presented in the Figure 4. | Figure 4 / 7-8 |
| | 20d | Presented | Results/6-8 |
| Reporting biases | 21 | Risk of bias assessment is shown in supplementary figure 2 and figure 3 | supplementary figure 2 & Figure 3 /8 |

# PRISMA 2020 Checklist

| Section and Topic | Item # | Checklist item | Location where item is reported / Page No. |
|---|---|---|---|
| Certainty of evidence | 22 | Not available | NA |
| DISCUSSION | | | |
| Discussion | 23a | Provide a general interpretation of the results in the context of other evidence.<br>Presented | Discussion / 9-11 |
| | 23b | Discuss any limitations of the evidence included in the review.<br>Presented | Discussion, last paragraph / 10-11 |
| | 23c | Discuss any limitations of the review processes used.<br>Presented | Discussion, last paragraph / 1ß-11 |
| | 23d | Discuss implications of the results for practice, policy, and future research.<br>Presented | Discussion / 9-11 |
| OTHER INFORMATION | | | |
| Registration and protocol | 24a | The study was registered in the "International Prospective Register of Systematic Reviews" (PROSPERO) in 2024 (Submission-ID: 507697) | Methods / 3 |
| | 24b | The study was registered in the "International Prospective Register of Systematic Reviews" (PROSPERO) in 2024 (Submission-ID: 507697) | Methods / 3 |
| | 24c | No amendments to describe | NA 7 11 |
| Support | 25 | There is no financial conflict of interest to declare | NA / 11 |
| Competing interests | 26 | No competing interests of the authors to delcare | NA / 11 |
| Availability of data, code and other materials | 27 | Template data collection form, data extracted and used for the analysis are reported in the section "methods". The R packages (IPDfromKM, SURVMINER, METAFOR, and SURVIVAL) are available online | Methods + Declarations/ 11 |

*From:* Page MJ, McKenzie JE, Bossuyt PM, Boutron I, Hoffmann TC, Mulrow CD, et al. The PRISMA 2020 statement: an updated guideline for reporting systematic reviews. BMJ 2021;372:n71. doi: 10.1136/bmj.n71

For more information, visit: http://www.prisma-statement.org/
