# Supplementary figures and images for: Impact of levetiracetam use in glioblastoma: an individual patient-level meta-analysis assessing overall survival
Source: Neurosurg Rev. 2024 Dec 9;47(1):897. doi: 10.1007/s10143-024-03137-x (PMC11628436; doi:10.1007/s10143-024-03137-x)

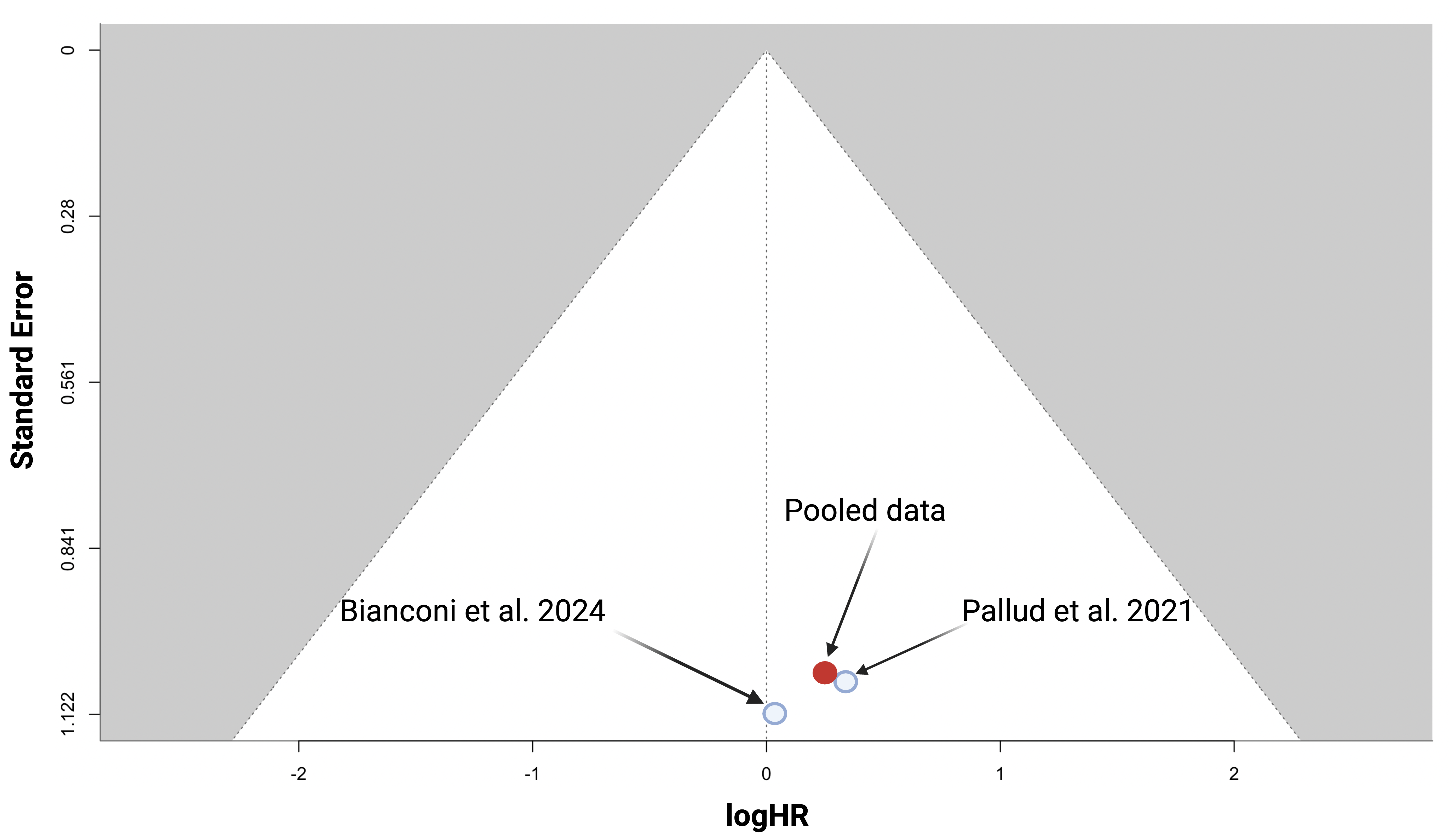

Supplement: Supplementary file 2 — Supplementary Material 2. [file 10143_2024_3137_MOESM2_ESM.tiff]

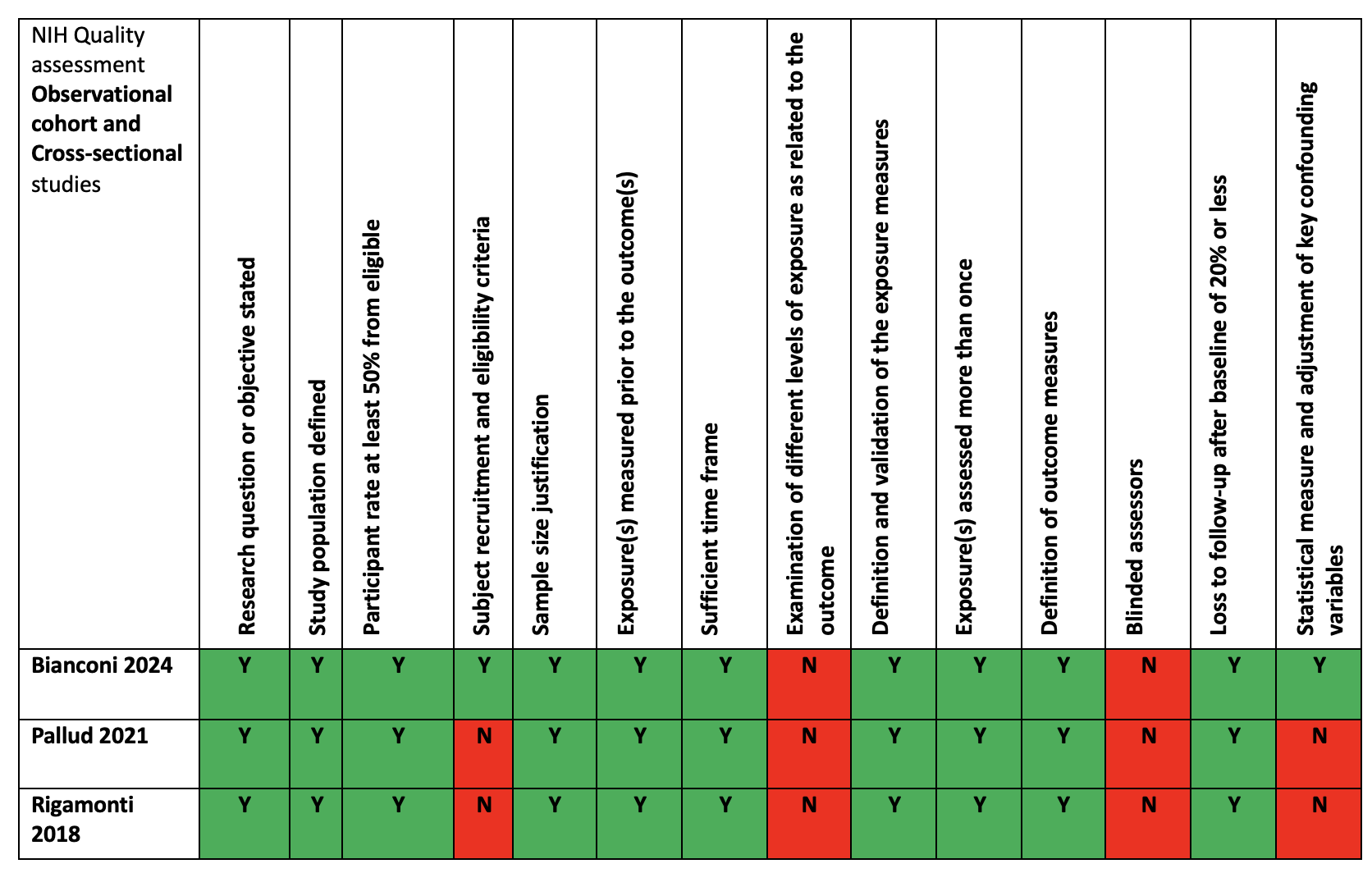

Supplement: Supplementary file 3 — Supplementary Material 3. [file 10143_2024_3137_MOESM3_ESM.tiff]

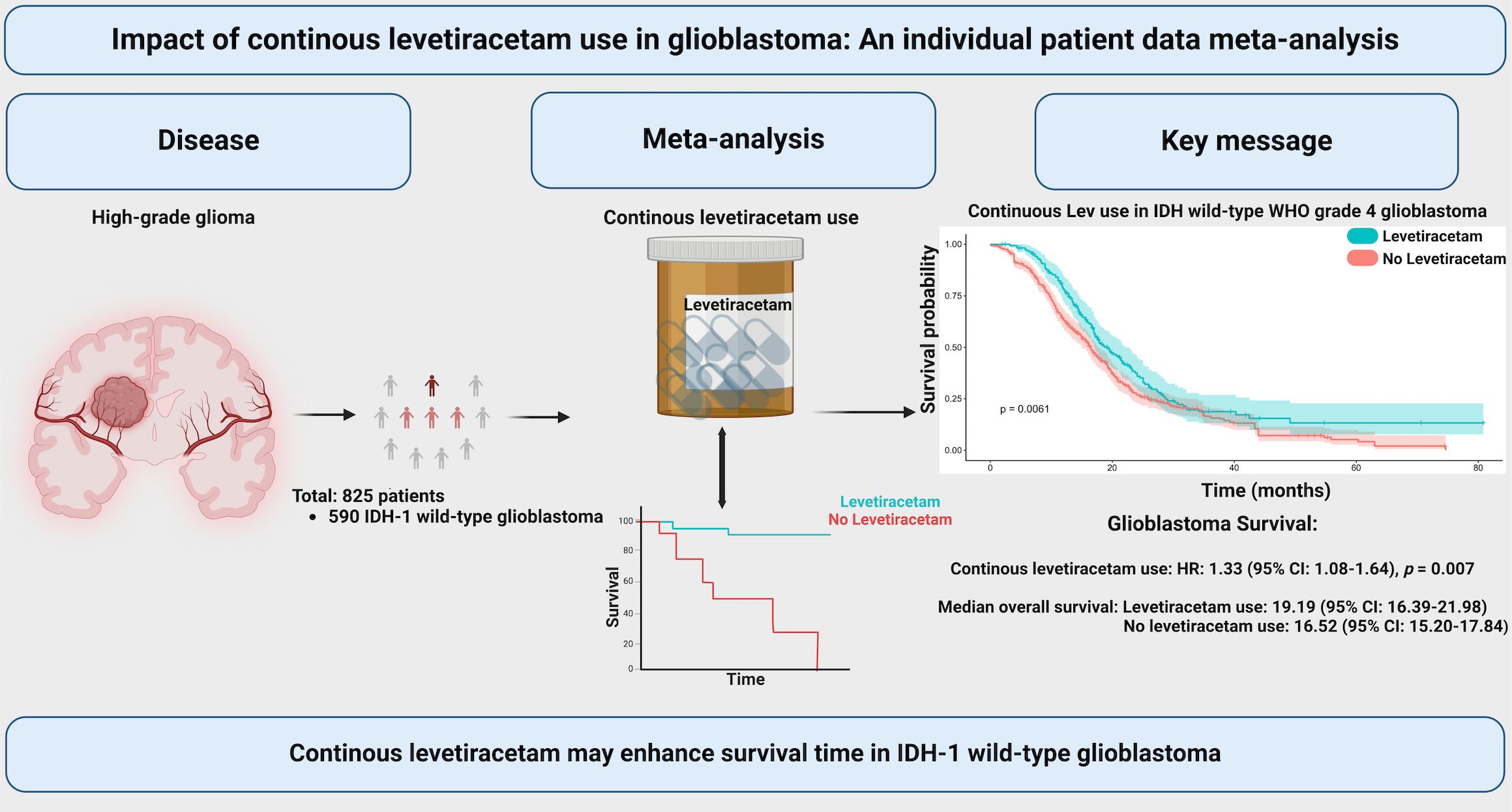

Supplement: Supplementary file 4 — Supplementary Material 4. [file 10143_2024_3137_MOESM4_ESM.tiff]

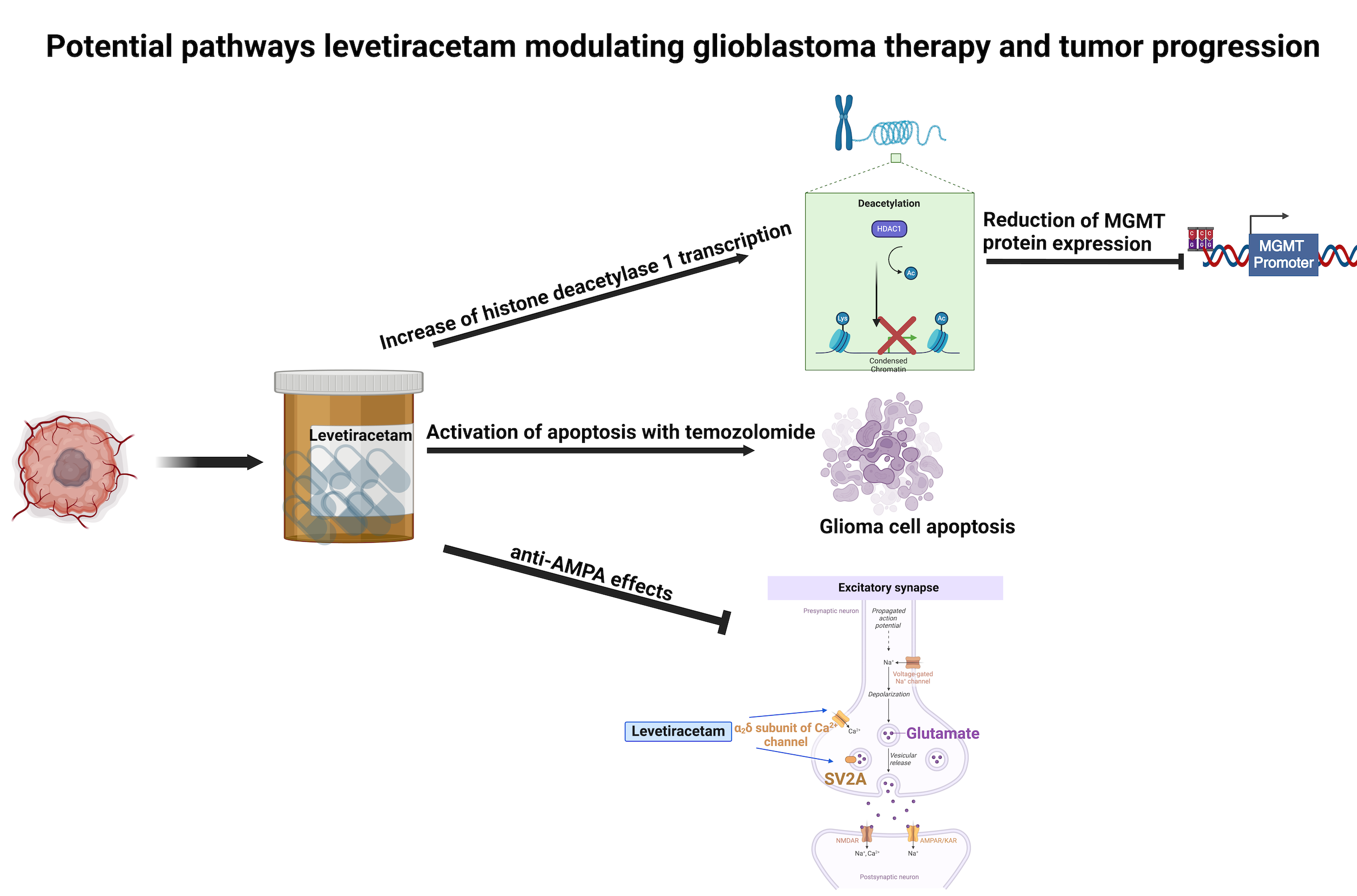

Supplement: Supplementary file 5 — Supplementary Material 5. [file 10143_2024_3137_MOESM5_ESM.tiff]
