# Supplementary material for: Impact of levetiracetam use in glioblastoma: an individual patient-level meta-analysis assessing overall survival
Source: Neurosurg Rev. 2024 Dec 9;47(1):897. doi: 10.1007/s10143-024-03137-x (PMC11628436; doi:10.1007/s10143-024-03137-x)
Supplement: Supplementary file 6 — Supplementary Material 6. [file 10143_2024_3137_MOESM6_ESM.docx]

| **Supplementary Table 1** Search Strategies Used for Systematic Review and Meta-Analysis in PubMed, EMBASE, Cochrane, and Medline Databases | | |
| --- | --- | --- |
| **S.no.** | **Database** | **Search syntax** |
| 1 | PubMed | ("glioblastoma" AND "levetiracetam") OR ("glioma" AND "levetiracetam") |
| 2 | EMBASE | ('glioblastoma'/exp OR glioblastoma OR 'glioma'/exp OR glioma) AND ('levetiracetam'/exp OR levetiracetam) AND ('systematic review'/exp OR 'meta analysis'/exp) |
| 3 | Cochrane | (glioblastoma OR glioma) AND levetiracetam AND review |
| 4 | Medline | ("glioblastoma" OR "glioma") AND ("levetiracetam") AND (review OR "meta-analysis") |
