# Supplementary material for: Impact of levetiracetam use in glioblastoma: an individual patient-level meta-analysis assessing overall survival
Source: Neurosurg Rev. 2024 Dec 9;47(1):897. doi: 10.1007/s10143-024-03137-x (PMC11628436; doi:10.1007/s10143-024-03137-x)
Supplement: Supplementary file 7 — Supplementary Material 7. [file 10143_2024_3137_MOESM7_ESM.docx]

| **Supplementary Table 2.** Cohort characteristics of included studies. | | | | | | |
| --- | --- | --- | --- | --- | --- | --- |
| **Study** | **Age (Years)** | **Male :**  **Female (n)** | **Surgery** | **MGMT-promotor methylation status** | **Therapy** | **Exclusion**  **criteria** |
| Bianconi et al. [20] | 63 | 167:105 | Resection only | 101 | Radiation + Temozolomide | Palliative care  Suspension of radiochemotherapy  Stereotactic biopsies  Patients under 18 years of age |
| Pallud et al. [21] | 60 | 270:190 | Biopsy (*n*=138)  Subtotal removal (*n*=105)  Total removal (*n*=217) | 104 | Radiation + Temozolomide | Patients under 18 years of age |
| Rigamonti et al. [22] | 67 | 178:107 | Biopsy (*n*=33)  Subtotal removal (*n*=55)  Total removal (*n*=197) | N/A | Radiation + Temozolomide  Radiation  No therapy | Patients under 18 years of age |
