# Supplementary material for: Impact of levetiracetam use in glioblastoma: an individual patient-level meta-analysis assessing overall survival
Source: Neurosurg Rev. 2024 Dec 9;47(1):897. doi: 10.1007/s10143-024-03137-x (PMC11628436; doi:10.1007/s10143-024-03137-x)
Supplement: Supplementary file 8 — Supplementary Material 8. [file 10143_2024_3137_MOESM8_ESM.docx]

| **Supplementary Table 3** Estimated Hazard ratios (HR) and corresponding 95% confidence intervals regarding progression-free survival in the one-stage meta-analysis for all high-grade gliomas | | |
| --- | --- | --- |
| **Study, year** | **Estimated HR** | **Estimated 95% CI** |
| Rigamonti et al., 2018 [22] | 1.20 | 0.89-1.62 |
| Pallud et al., 2021 [21] | 1.40 | 1.10-1.78 |
| Bianconi et al., 2024 [20] | 1.02 | 0.65-1.61 |
| Entire IPD cohort (IDH mutant high-grade glioma & IDH wild-type GB) | 1.20 | 1.02-1.42 |
